# NUPR1 in breast cancer: mechanisms and potential applications

**DOI:** 10.3389/fphys.2026.1880388

**Published:** 2026-07-09

**Authors:** Bo Xiang, Tao Liu, Duo Xu, Wei Wang

**Affiliations:** Department of Breast Surgery, Jilin Cancer Hospital, Changchun, China

**Keywords:** autophagy, breast cancer, ferroptosis, metabolic reprogramming, NUPR1, therapeutic resistance

## Abstract

Breast cancer continues to present formidable clinical challenges, particularly in triple-negative and endocrine-resistant subtypes where adaptive stress mechanisms drive therapeutic failure. Nuclear protein 1 (NUPR1), an intrinsically disordered protein, has emerged as a non-mutational hub that has been implicated in integrating metabolic, transcriptional, and cell-survival signals associated with malignant progression. This Review examines how NUPR1 transduces mitogenic stimuli into anabolic programs, while orchestrating autophagic flux, lysosomal biogenesis, and ferroptosis evasion to maintain cellular fitness under oncogenic and therapeutic stress. We discuss its causal roles in endocrine and chemoresistance through chromatin-associated cooperation with estrogen receptor α, activation of DNA-damage repair, and cell-cycle checkpoint control, as well as its contributions to metastatic dissemination via extracellular vesicle-mediated niche remodeling and immunosuppressive macrophage polarization. Furthermore, we evaluate emerging therapeutic avenues, from small-molecule inhibitors and single-domain antibody degraders that disrupt NUPR1 nuclear trafficking, to metabolic drug repurposing strategies such as statins that intercept the insulin–NUPR1 axis. Elucidating NUPR1 biology represents a paradigm shift toward targeting dynamic, stress-adaptive dependencies in breast cancer, offering new precision-oncology opportunities.

## Introduction

Breast cancer (BC) remains the most frequently diagnosed malignancy among women worldwide and a leading cause of cancer-related mortality (Chen et al., 2026; Cho et al., 2026). While advances in early detection, molecular subtyping, and targeted therapies, including endocrine agents, anti-HER2 monoclonal antibodies, and immune checkpoint inhibitors, have transformed the clinical landscape, substantial challenges persist (Li et al., 2021; Shin et al., 2026; Stachyra et al., 2026). A significant proportion of patients, particularly those with triple-negative breast cancer (TNBC) or metabolically dysregulated ER-positive disease, develop therapeutic resistance or relapse (Kim and Lukong, 2026; López-Santana et al., 2026; Lobo-Martins et al., 2026). Emerging evidence underscores that metabolic perturbations, such as obesity-associated hyperinsulinemia and hypercholesterolemia, not only increase BC risk but also fuel tumor progression and compromise treatment efficacy (Guo and Addison, 2026; Marouf et al., 2026; Ruggeri et al., 2026; Wang et al., 2026). Consequently, there is an urgent need to dissect the stress-adaptive mechanisms that enable BC cells to survive metabolic insults and therapeutic pressure, and to identify druggable nodes within these pathways.

Nuclear protein 1 (NUPR1) has emerged as a central, non-mutational hub in this adaptive repertoire (Carracedo et al., 2006a; Estaras et al., 2025). Originally identified as a stress-inducible factor in acute pancreatitis, NUPR1 is an intrinsically disordered protein (IDP) of approximately 8.8 kDa that lacks stable secondary or tertiary structures (Kim et al., 2026; Long et al., 2026). This biophysical property allows NUPR1 to function as a versatile transcriptional co-regulator and chromatin-associated factor, forming transient complexes with diverse molecular partners in response to cellular stress (Scordamaglia et al., 2026). By binding AT-rich promoter regions and indirectly modulating key signaling mediators, including NF-κB, SMAD, and PI3K/AKT, NUPR1 orchestrates a broad spectrum of biological outputs: metabolic reprogramming, proteostatic control, autophagic flux, and cell-fate decisions that restrain apoptosis and ferroptosis (Tong et al., 2022; Deng et al., 2026; Luo et al., 2026; Luo et al., 2026; Yu et al., 2026).

In the context of breast cancer, NUPR1 has recently been positioned at the nexus of metabolic signaling and therapeutic response (Wang et al., 2025; Scordamaglia et al., 2026). Mechanistically, NUPR1 is induced by mitogenic stimuli such as insulin and functions downstream of the insulin receptor (IR)–IRS1–AKT signaling cascade to promote proliferation, clonogenic capacity, and spheroid expansion (Clark et al., 2008; Long et al., 2026). Notably, pharmacological inhibition of NUPR1, either by small-molecule antagonists such as ZZW-115 or by novel single-domain antibodies such as sdAb#07.81, blunts insulin-driven oncogenic signaling, derepresses ferroptosis, and induces premature senescence, thereby suppressing tumor growth in preclinical models (Lim et al., 2024). Furthermore, NUPR1 drives resistance to conventional modalities: in endocrine therapy, it cooperates with estrogen receptor α (ESR1) to sustain autophagic flux and maintain tamoxifen resistance; in chemotherapy, it activates anti-apoptotic and DNA-repair programs; and in the tumor microenvironment, it contributes to immunosuppressive macrophage polarization (Lan et al., 2020; Wang et al., 2021; Tan et al., 2024; Wang et al., 2025; Liu et al., 2026; Scordamaglia et al., 2026).

**Figure 1:**
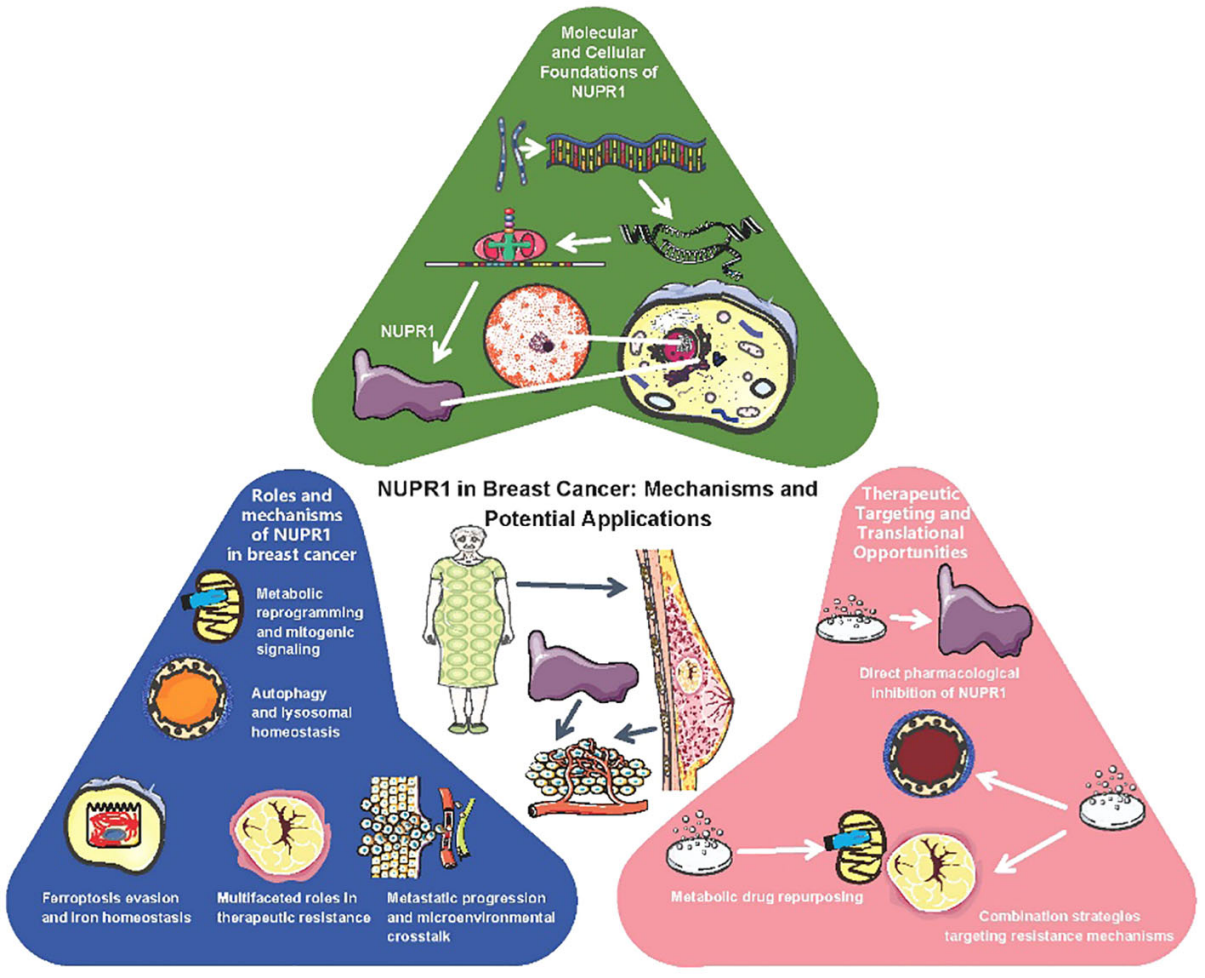
Molecular and cellular foundations of NUPR1 and roles of NUPR1 in breast cancer. Parts of **Figure 1** were drawn using pictures from Servier Medical Art. Servier Medical Art by Servier is licensed under a Creative Commons Attribution 3.0 Unported License (https://creativecommons.org/licenses/by/3.0/)

Despite this progress, critical questions remain unresolved. The functional divergence between NUPR1 isoforms, the context-dependent nature of its regulatory networks across BC subtypes, and the optimal strategies for integrating NUPR1 inhibition into existing treatment paradigms require comprehensive evaluation. In this Review, we synthesize the molecular and cellular foundations of NUPR1 biology, dissect its multifaceted roles in breast cancer initiation, progression, and therapy resistance, and discuss emerging therapeutic targeting strategies, from small-molecule inhibitors and antibody-based degraders to metabolic drug repurposing, that aim to convert NUPR1 from an adaptive stress node into a clinically actionable vulnerability (**Figure 1**).

## Molecular and cellular foundations of NUPR1

### Gene organization, isoforms and protein architecture

The human *NUPR1* gene (also known as *p8* or *COM1*) resides on chromosome 16p11.2 and comprises three exons and two introns (Ree et al., 1999; Bratland et al., 2000; Igarashi et al., 2001). It encodes two distinct protein isoforms through alternative splicing: the canonical NUPR1b isoform of 82 amino acids (~8.8 kDa) and the longer NUPR1a isoform of 100 residues, which contains an additional 18 amino acids of poorly defined function (Encinar et al., 2001; Hoffmeister et al., 2002; Ito et al., 2003). Almost all current biochemical and cancer-biology studies have focused on NUPR1b, whereas the spatiotemporal expression, structural behaviour and pathophysiological contribution of NUPR1a remain largely unexplored, a knowledge gap that is particularly relevant given emerging evidence of isoform-specific interactomes in breast cancer cells. To systematically disentangle these isoform-specific functions, future studies could deploy high-dimensional approaches, including bulk and single-cell RNA-seq splice-variant analysis, isoform-specific affinity purification coupled to mass spectrometry (AP-MS), and isoform-selective proximity labeling, to map NUPR1a-specific expression patterns, protein–protein interaction networks, and chromatin occupancy across breast cancer molecular subtypes. Because the overwhelming majority of published functional and cancer-biology datasets focus exclusively on the canonical 82-amino-acid variant, the designation ‘NUPR1’ is utilized throughout the remainder of this Review to denote the NUPR1b isoform, unless specified otherwise.

NUPR1b is a highly basic protein organized into three functional segments (Mallo et al., 1997; Igarashi et al., 2001; Grasso et al., 2015; Lopez et al., 2015). The N-terminal region harbours a proline-, glutamic acid-, serine- and threonine-rich (PEST) sequence that targets the protein for ubiquitin–proteasome–dependent degradation and links its turnover to phosphatidylinositol 3-kinase (PI3K)/AKT signalling (Carracedo et al., 2006b). The C terminus contains a classical bipartite nuclear localization signal (NLS; residues ~63–78) superimposed on a basic helix–loop–helix (HLH) motif (Valacco et al., 2006; Qiu and MacRae, 2007; Wu et al., 2022). Within this region, two conserved acetylation and methylation sites modulate electrostatic interactions with nuclear transport machinery; acetylation of these lysines is essential for efficient nuclear import, whereas phosphorylation of Thr68 within the NLS can impede binding to importin-α family members and thereby restrict nuclear retention (Valacco et al., 2006; Santofimia-Castaño et al., 2017; Santofimia-Castaño et al., 2022). This architectural arrangement, an unstable N-terminal degradation signal coupled to a C-terminal DNA-binding and trafficking module, endows NUPR1 with rapid signal responsiveness and tight spatial control.

### Intrinsically disordered nature and conformational dynamics

A defining feature of NUPR1 is that it belongs to the family of intrinsically disordered proteins (IDPs) (Neira et al., 2018; Neira et al., 2019). Biophysical analyses by circular dichroism, Fourier-transform infrared spectroscopy, fluorescence and NMR have failed to detect stable secondary or tertiary structure under native conditions; the protein behaves as an extended, highly flexible polypeptide in both dilute solution and crowded environments that mimic the intracellular milieu (Santofimia-Castaño et al., 2020; Santofimia-Castaño et al., 2021; Araujo-Abad et al., 2023). This extreme conformational plasticity is not a structural deficit but rather a functional asset. Like many IDPs, NUPR1 operates through dynamic complexes: it maintains its disordered state even when bound to DNA or to protein partners such as MSL1, PARP1, PKP1 and PADI4, allowing a single molecule to engage multiple, structurally diverse interactors with high specificity yet rapid reversibility (Ghandhi et al., 2014; Santofimia-Castaño et al., 2022; Araujo-Abad et al., 2023).

Phosphorylation by protein kinase A (PKA) introduces a partial ordering of the polypeptide chain and significantly enhances DNA-binding affinity, suggesting that post-translational modifications can shift NUPR1 from a transcriptionally inert, highly disordered state to a more structured, chromatin-associated conformation (Encinar et al., 2001). In the context of breast cancer, this phosphorylation-dependent switch may couple mitogenic kinase signalling to NUPR1-mediated transcriptional programmes (Marouf et al., 2026). Moreover, the disordered nature of NUPR1 predisposes it to liquid–liquid phase separation (LLPS), a phenomenon increasingly recognized in RNA-binding proteins and transcriptional hubs (Santofimia-Castaño et al., 2024; Xu et al., 2024). Recent work demonstrates that NUPR1 drives the assembly of stress granules, membraneless cytoplasmic organelles that sequester mRNA and translational machinery under oncogenic or metabolic stress, thereby reprogramming protein synthesis and fostering survival of KRAS-mutant and triple-negative breast cancer cells (Santofimia-Castaño et al., 2024; Schneider and Schneider, 2024; Álvarez-Rodríguez et al., 2026).

### Subcellular trafficking and nucleo-cytoplasmic shuttling

NUPR1 shuttles between the nucleus and cytoplasm, and its subcellular distribution is context- and pathology-dependent (Vasseur et al., 1999). In normal thyroid follicles and renal tubules, NUPR1 is predominantly nuclear, consistent with its role as a chromatin-associated factor (Ito et al., 2003; Ito et al., 2005). By contrast, pronounced cytoplasmic staining is observed in poorly differentiated, lymph node–positive breast tumours and in non-small cell lung cancers, suggesting that oncogenic progression may derail nuclear import or accelerate nuclear export (Ito et al., 2003; Päth et al., 2006; Li et al., 2024). In breast cancer cell lines, NUPR1 has been detected in both compartments, and its cytoplasmic retention correlates with metastatic potential (Ozkaya et al., 2017; Lim et al., 2024; Ortiz et al., 2024).

Nuclear import is mediated by the NLS-dependent recognition of importin-α family members (Valacco et al., 2006; Santofimia-Castaño et al., 2019b). The small-molecule inhibitor ZZW-115 exploits this trafficking dependency: by binding to residues Ala33 and Thr68, it competitively disrupts the NUPR1–importin interaction, blocks nuclear translocation and traps NUPR1 in the cytoplasm (Lan et al., 2020). Because Thr68 lies within the NLS, phosphorylation at this site also hampers importin binding, providing a potential regulatory checkpoint that links upstream kinase signaling to NUPR1 localization (Neira et al., 2020; Estaras et al., 2025). Cytoplasmic sequestration not only abrogates NUPR1’s nuclear transcriptional functions but also unmasks cytoplasmic roles, such as stress-granule scaffolding and modulation of autophagic machinery, that may become dominant in specific breast cancer subtypes (Wu et al., 2020; Wang et al., 2021; Scordamaglia et al., 2026).

### Transcriptional regulatory mechanisms and chromatin association

NUPR1 functions as a transcriptional co-regulator rather than a classical sequence-specific transcription factor (Vasseur et al., 1999; Encinar et al., 2001). It binds preferentially to AT-rich promoter regions and indirectly modulates downstream pathways by recruiting or stabilizing key signaling mediators (Vasseur et al., 2004; Giroux et al., 2006). In breast cancer cells, NUPR1 has been shown to cooperate with NF-κB, SMAD and PI3K/AKT signaling to regulate genes involved in autophagy, lysosomal biogenesis and cell-cycle control (Wang et al., 2021; Aguilar et al., 2023; Lee et al., 2025; Wang et al., 2025; Liu et al., 2026).

Beyond direct DNA contact, NUPR1 participates in DNA-damage repair through its interaction with MSL proteins (Gironella et al., 2009). Upon double-strand breaks, NUPR1 is recruited to chromatin, where it forms a dose-dependent complex with MSL1 that facilitates DNA-repair activity and protects against genotoxic cell death (Aguado-Llera et al., 2013). In addition, NUPR1 binds PARP1 in the nucleus and restrains its enzymatic activity under homeostatic conditions (Tang et al., 2015; Santofimia-Castaño et al., 2022). These observations position NUPR1 as a chromatin-associated hub that integrates transcriptional control with genome-maintenance programs.

### Post-translational modifications and functional modulation

NUPR1 function is tightly governed by post-translational modifications that modulate its stability, localization and interactome (Garbacki et al., 2023; Zhao et al., 2026). Ubiquitination of the PEST domain targets NUPR1 for proteasomal degradation, a process regulated by PI3K/AKT; this provides a direct mechanistic link between growth-factor signaling and NUPR1 abundance (Sárvári et al., 2010). Acetylation within the C-terminal HLH/NLS region promotes nuclear import and chromatin engagement, whereas methylation at adjacent residues may fine-tune DNA-binding affinity (Neira et al., 2020). Phosphorylation, particularly at Thr68, introduces a conformational switch that both enhances DNA binding and restricts nuclear import, creating a feedback loop that adjusts NUPR1 activity to cellular kinase status (Neira et al., 2018; Santofimia-Castaño et al., 2019b).

In breast cancer cells, these modifications are frequently hijacked by oncogenic pathways. For example, HDAC11 downregulation in doxorubicin-resistant triple-negative breast cancer leads to hyperacetylation of the NUPR1 promoter and relieves transcriptional repression, resulting in NUPR1 overexpression and chemoresistance (Lim et al., 2024). Conversely, pharmacological inhibition of NUPR1 by ZZW-115 or trifluoperazine derivatives blocks acetylation-dependent nuclear trafficking and disrupts the SUMOylation of DNA-damage-response proteins, shifting the cellular balance from survival to ferroptosis, apoptosis or necroptosis (Lan et al., 2020; Huang et al., 2021b).

## Roles and mechanisms of NUPR1 in breast cancer

**Figure 2:**
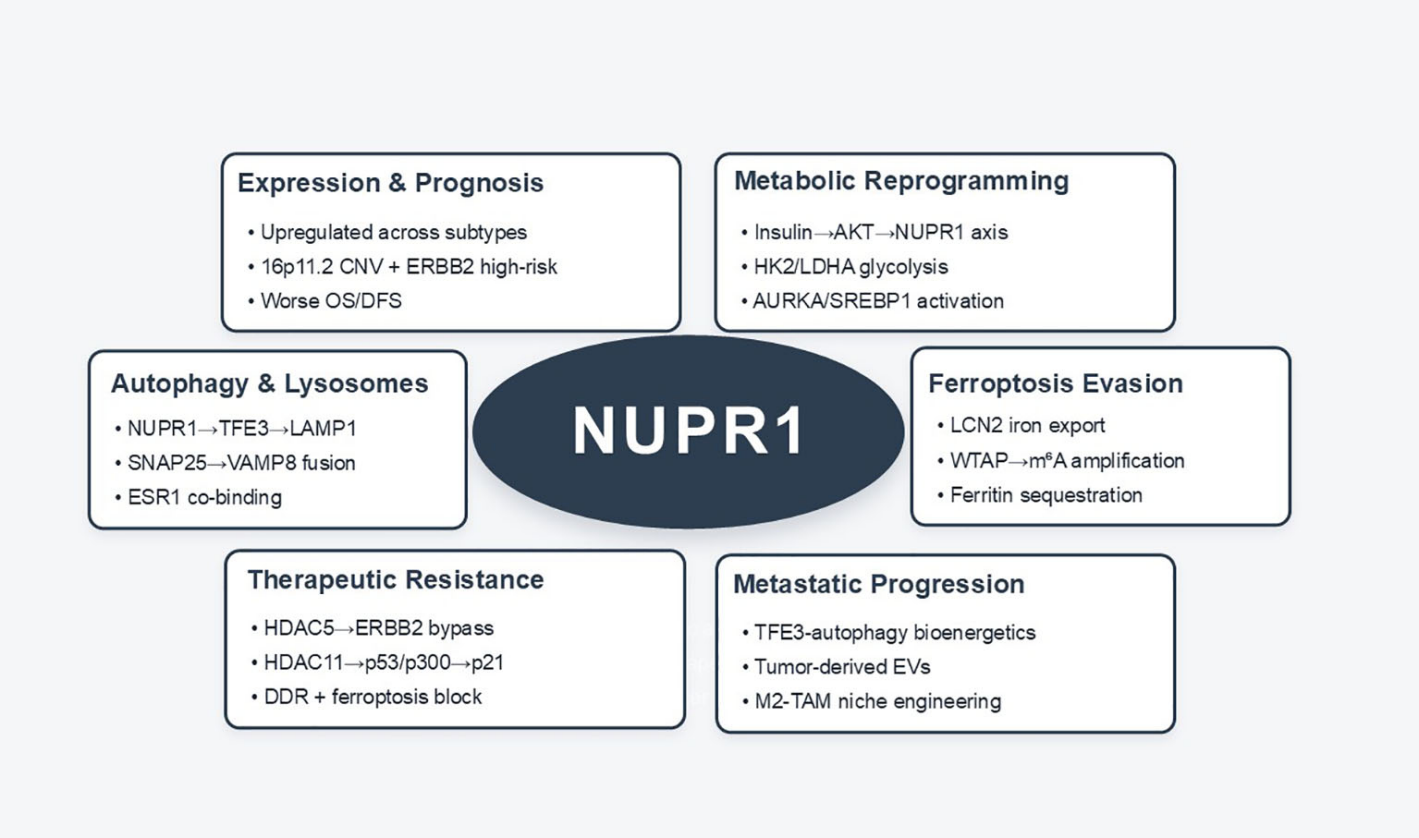
Roles and mechanisms of NUPR1 in breast cancer: metabolic reprogramming and mitogenic signaling.

Building upon the molecular and architectural features outlined above, NUPR1 functions as a pleiotropic, stress-adaptive hub that is systematically co-opted by breast cancer cells to drive malignant transformation, metabolic plasticity, therapeutic evasion, and metastatic dissemination. In this section, we synthesize the current evidence positioning NUPR1 at the center of breast cancer pathobiology, dissecting its subtype-specific roles and the mechanistic circuits that link its intrinsically disordered biology to clinical aggressiveness (**Figure 2**).

### Expression, genomic alterations and prognostic significance

NUPR1 is markedly upregulated across multiple breast cancer subtypes compared with normal mammary epithelium (Bratland et al., 2000; Ree et al., 2000; Wang et al., 2021; Wu et al., 2022; Aguilar et al., 2023). The transcriptomic analyses of breast cancer cohorts demonstrate that elevated NUPR1 expression is associated with significantly worse overall survival and shortened disease-free intervals (Jiang et al., 2005; Jung et al., 2012). These prognostic associations are not limited to luminal disease; Kaplan–Meier analyses further reveal that high NUPR1 mRNA levels correlate with poor overall and relapse-free survival in both endocrine therapy-treated ER-positive and ERBB2-enriched breast cancer populations (Jiang et al., 2005; Jung et al., 2012; Lee et al., 2025). At the genomic level, copy-number gain at 16p11.2, which harbors the NUPR1 locus, represents one of the recurrently altered regions in early-stage breast cancer (Jung et al., 2012). Notably, simultaneous copy-number gains of NUPR1 (16p11.2) and ERBB2 (17q12) define a high-risk genotypic configuration that predicts particularly poor prognosis in early-stage disease (Jung et al., 2012). This genomic co-alteration suggests that NUPR1-driven transcriptional programs may synergize with ERBB2 signaling to accelerate tumor progression. More recently, single-cell transcriptomics and machine-learning-based glycolytic scoring have identified NUPR1 as one of seven core glycolytic regulatory hub genes in breast cancer, with its expression enriched in malignant epithelial subsets exhibiting the highest glycolytic activity (Liu et al., 2026). Collectively, these clinical and genomic data establish NUPR1 not merely as a passenger alteration but as a bona fide biomarker of aggressive disease biology.

### Metabolic reprogramming and mitogenic signaling

NUPR1 has been positioned as a contributor to metabolic adaptation in breast cancer, with evidence suggesting it may function as a bidirectional transducer that links extracellular mitogenic cues to intracellular anabolic programs (Liu et al., 2026; Scordamaglia et al., 2026). Recent single-cell transcriptomic analyses integrating machine-learning-based glycolytic scoring have identified NUPR1 as one of seven core glycolytic regulatory hub genes in breast cancer, with its expression selectively enriched in malignant epithelial subsets exhibiting the highest glycolytic activity (Mu et al., 2018; Liu et al., 2026).

In the context of obesity-associated breast cancer, NUPR1 is robustly induced by insulin stimulation and appears to operate as an effector of the insulin receptor (IR)–IRS1–AKT signaling cascade in both ER-positive and triple-negative models (Päth et al., 2020; Lagarde et al., 2024; Marouf et al., 2026; Scordamaglia et al., 2026). Mechanistically, PI3K/AKT signaling stabilizes NUPR1 protein by suppressing ubiquitin–proteasome degradation through its N-terminal PEST domain, establishing a positive feed-forward loop that amplifies oncogenic output (Li et al., 2020). Functionally, NUPR1 activation enhances proliferative capacity, clonogenic survival, and spheroid expansion in response to insulin. The spheroid phenotype is particularly informative in this context. Unlike standard two-dimensional (2D) monolayer cultures, in which cells experience homogeneous oxygen and nutrient availability, three-dimensional (3D) spheroids establish steep spatial gradients of oxygen deprivation, nutrient limitation, and metabolite accumulation that closely recapitulate the avascular microregions of solid breast tumors (Boot et al., 2021; Quoniou and Menecier, 2026). This distinction carries critical translational implications: preclinical efficacy of NUPR1 inhibitors assessed solely in 2D monolayers may underestimate the tumor dependency on NUPR1-driven stress adaptation that manifests *in vivo*.

Beyond its role as an insulin effector, NUPR1 actively reshapes glucose metabolism. Under mitochondrial dysfunction or calcium overload, which are common in rapidly proliferating breast cancer cells, NUPR1 transcription is markedly upregulated (Syn et al., 2017; Iovanna et al., 2025). In hepatocellular carcinoma models, NUPR1 binds to the promoter region of the GRN gene, thereby activating PI3K/AKT and MAPK signaling pathways (Lee et al., 2015). This transcriptional relay upregulates the key glycolytic enzymes hexokinase 2 (HK2) and lactate dehydrogenase A (LDHA), redirecting glucose flux from oxidative phosphorylation toward aerobic glycolysis (Zhao et al., 2026). Beyond bioenergetic and biosynthetic consequences, this glycolytic switch fundamentally alters cellular redox poise. By maintaining rapid LDHA-dependent NAD regeneration, NUPR1-driven aerobic glycolysis redirects pyruvate flux away from mitochondrial oxidative phosphorylation. This restriction of substrate delivery to the tricarboxylic acid cycle reduces electron congestion across respiratory complexes I and III, thereby attenuating basal mitochondrial ROS generation and preserving the cellular pools of NADPH required to sustain glutathione-dependent defences against ferroptotic lipid peroxidation (Huang et al., 2021a; Liu et al., 2025; Zhao et al., 2026). The resultant lactate accumulation not only fuels biomass synthesis but also acidifies the microenvironment, fostering immune evasion and metastatic competency (Liu et al., 2025; Zhao et al., 2026). While the mitogen-driven insulin receptor-IRS1-AKT-NUPR1 signaling cascade is empirically validated across both luminal and triple-negative breast cancer models, downstream metabolic branches such as the GRN-HK2/LDHA axis remain to be confirmed within mammary systems.

Importantly, this metabolic configuration generates a paradoxical redox vulnerability. While glycolytic suppression of mitochondrial ROS is protective under basal conditions, the concomitant accumulation of lactate and protons acidifies the cytosol and can exacerbate oxidative stress during nutrient fluctuations or therapeutic insult (Liu et al., 2025; Zhao et al., 2026). We posit that NUPR1 does not merely promote glycolysis for anabolic gain, but actively constructs a redox buffer zone that anticipates and counteracts oxidative membrane damage, a function that converges directly on its downstream ferroptosis-suppressive activity via LCN2 and ferritin iron sequestration (Narzt et al., 2019; Huang et al., 2021a; Zhan et al., 2022; Long et al., 2026).

By analogy to stress-response mechanisms characterised in pancreatic adenocarcinoma, NUPR1 is hypothesised to engage an alternative survival module under energy-limiting conditions by activating Aurora kinase A (AURKA). While this NUPR1-AURKA axis transcriptionally coordinates DNA repair and cell-cycle progression in non-mammary settings (Hamidi et al., 2012; Kaya et al., 2022), its functional relevance to breast cancer metabolic adaptation remains purely speculative and awaits formal experimental validation.

Although most prominent in hepatocellular carcinoma, NUPR1–SREBP1 lipogenic axis may also be relevant in aggressive breast cancer subtypes (Wang et al., 2022). NUPR1 enhances nuclear translocation of mature SREBP1 and enriches at lipogenic gene promoters, driving *de novo* fatty acid synthesis (Wang et al., 2022). Intriguingly, widely prescribed metabolic agents such as statins indirectly impinge upon this network (Scordamaglia et al., 2026). By inhibiting HMG-CoA reductase and the mevalonate pathway, statins impair IR-mediated signaling and downregulate NUPR1 expression, thereby attenuating insulin-stimulated proliferation and clonogenicity in ER-positive breast cancer cells (Alim and Akalanka, 2026). These findings provide a mechanistic rationale for repurposing lipid-lowering drugs in tumors characterized by hyperactive insulin signaling and elevated NUPR1.

### Autophagy and lysosomal homeostasis

Autophagy is a central survival mechanism through which breast cancer cells endure metabolic insults, genotoxic stress, and therapeutic pressure (Zhou et al., 2023; Panicker et al., 2026; Rui et al., 2026). NUPR1 has been implicated as a regulator that influences autophagy at multiple stages, from lysosomal biogenesis to autophagosome–lysosome fusion and cargo clearance, and may promote the shift of autophagy from a homeostatic degradation process toward a pro-survival, pro-metastatic phenotype (Fan et al., 2022; Xiao et al., 2022; Ren et al., 2024).

NUPR1 is associated with increased autophagy and transcriptional activation of transcription factor E3 (TFE3), a key regulator of lysosomal biogenesis and autophagy gene expression (Wang et al., 2024). In breast cancer cells, reports suggest that NUPR1 may bind to the TFE3 promoter and promote expression of lysosomal genes such as LAMP1, enhancing lysosomal acidification and proteolytic capacity (Xiao et al., 2022). NUPR1 suppression reduces TFE3 levels, lowers LAMP1 expression, diminishes lysosomal proteolysis, and ultimately blocks autophagic flux (Xiao et al., 2022). Exogenous TFE3 rescues lysosomal activity and reduces accumulation of LC3B-II and p62 in NUPR1-deficient cells, confirming that the NUPR1–TFE3 axis is indispensable for maintaining lysosomal homeostasis (Xiao et al., 2022). From a redox perspective, this lysosomal maintenance is critical because impaired autophagic flux leads to the accumulation of dysfunctional mitochondria and protein aggregates that generate ROS and lipid peroxides. By sustaining TFE3-driven lysosomal capacity, NUPR1 ensures efficient clearance of oxidatively damaged organelles, thereby suppressing the ROS buildup that constitutes a prerequisite for ferroptotic execution (Xiao et al., 2022; Wang et al., 2024; Long et al., 2026). This mechanism operates in breast, cervical, and oral squamous cell carcinomas, indicating a broadly conserved protumorigenic pathway. While the NUPR1–TFE3–LAMP1 and NUPR1–SNAP25–VAMP8 autophagic axes are directly validated in breast cancer cell lines and xenografts, the broader hypothesis that NUPR1 uniformly converts autophagy into a dedicated pro-metastatic engine across all breast cancer subtypes awaits wider subtype-specific validation.

The NUPR1–SNAP25–VAMP8 axis controls autophagosome–lysosome fusion. Beyond initiating lysosomal programs, NUPR1 governs the terminal stages of autophagy (Mu et al., 2018; Yang et al., 2025). NUPR1 upregulates the synaptic-associated SNARE protein SNAP25, which complexes with vesicle-associated membrane protein 8 (VAMP8) to form the SNARE machinery that mediates autophagosome–lysosome fusion (Yang et al., 2025). In breast cancer models, NUPR1 silencing induces autophagosome accumulation, impairs mitophagy, and precipitates premature senescence (Mu et al., 2018). Notably, SNAP25 cooperates with the PTEN-induced kinase 1 (PINK1)/E3 ubiquitin ligase Parkin pathway to enhance mitophagy, thereby clearing damaged mitochondria and maintaining metabolic homeostasis (Cui et al., 2024; Guo et al., 2026). By simultaneously promoting lysosomal biogenesis and autophagosome maturation, NUPR1 ensures efficient autophagic flux from initiation to cargo degradation (Mu et al., 2018).

In tamoxifen-resistant ER-positive breast cancer, NUPR1 physically interacts with estrogen receptor α (ESR1) and binds to the promoter regions of autophagy and drug-resistance genes, including BECN1, GREB1, RAB31, PGR, and CYP1B1, to sustain autophagic flux (Wang et al., 2021). This NUPR1–ESR1 transcriptional complex prevents senescence and apoptosis under endocrine deprivation (Elango et al., 2021; Wang et al., 2021). Disruption of this complex, either by NUPR1 depletion or by pharmacological inhibition, raises lysosomal pH, impairs autophagic clearance, and induces premature senescence both *in vitro* and in xenograft models (Bertolini et al., 2020; Wang et al., 2021). Thus, NUPR1 converts autophagy from a homeostatic program into a dedicated resistance mechanism.

### Ferroptosis evasion and iron homeostasis

Ferroptosis, an iron-dependent form of regulated cell death driven by lethal lipid peroxidation, has emerged as a critical therapeutic vulnerability in triple-negative breast cancer (TNBC), which exhibits high iron dependence and limited apoptotic plasticity (Iftikhar et al., 2026; Lin et al., 2026; Liu et al., 2026). NUPR1 has been identified as a repressor of ferroptosis in select breast cancer subtypes, operating through a multi-pronged iron-control network that appears to limit both free iron availability and lipid peroxide accumulation (Liu et al., 2021; He et al., 2025; Zeng et al., 2026).

At the primary level, NUPR1 suppresses ferroptosis through direct transcriptional control of iron export. A proposed mechanism by which NUPR1 suppresses ferroptosis involves transcriptional upregulation of lipocalin-2 (LCN2), a secreted glycoprotein that sequesters iron-loaded siderophores and promotes cellular iron efflux (Luo et al., 2026). In TNBC cells, evidence suggests that NUPR1 binds directly to the LCN2 promoter, contributing to an iron-export program that diminishes intracellular Fe^2+^ pools and suppresses Fenton-catalyzed lipid peroxidation (Tan et al., 2024). Functional studies demonstrate that LCN2 depletion precisely phenocopies NUPR1 deficiency with respect to ferroptosis induction, whereas enforced re-expression of LCN2 fully restores ferroptosis resistance in NUPR1-knockdown cells (Tan et al., 2024).

This primary transcriptional output is further amplified by an epitranscriptomic mechanism. In TNBC, NUPR1 expression and its ferroptosis-suppressive output are further amplified by the m^6^A methyltransferase WTAP (Tan et al., 2024). WTAP promotes NUPR1 expression in an m^6^A–eIF3A-dependent manner: m^6^A modification of the NUPR1 transcript enhances its stability and translation efficiency, while eIF3A facilitates ribosomal loading (Tan et al., 2024). Consequently, the WTAP–NUPR1–LCN2 cascade creates a robust epitranscriptomic–transcriptional relay that locks TNBC cells into a ferroptosis-resistant state. Silencing of WTAP or pharmacologic inhibition of the m^6^A writer complex phenocopies NUPR1 loss, sensitizing TNBC cells to ferroptosis-inducing agents (Tan et al., 2024).

Beyond LCN2-driven iron export, NUPR1 also limits ferroptosis by promoting iron storage (Zhang et al., 2024), although the complementary FTH1/ferritin iron-sequestration layer remains putative in this context, as direct NUPR1 occupancy at the FTH1 promoter and functional validation in breast cancer systems are currently lacking. By promoting Fe^2+^ sequestration within ferritin cores, NUPR1 further reduces the labile iron pool available for Fenton chemistry, providing a complementary layer of protection against oxidative membrane damage (Isyraqiah et al., 2018; Zhang et al., 2024). The circPIAS1/miR-455-3p/NUPR1/FTH1 regulatory axis, first characterized in hepatocellular carcinoma, represents a modular circuit likely operative in aggressive breast cancer subtypes, wherein NUPR1 serves as the convergent transcriptional hub linking competing endogenous RNA networks to iron homeostasis (Zhang et al., 2024; He et al., 2025).

### Multifaceted roles in therapeutic resistance

NUPR1 is not merely a passive correlate of aggressive disease but appears to contribute to therapeutic resistance across endocrine, cytotoxic, and metabolic modalities (Botto et al., 2018; Yu et al., 2018). Its resistance functions operate through three interconnected layers: (i) epigenetic and transcriptional rewiring that alters drug-target expression and bypasses pathway blockade; (ii) cell-cycle and apoptotic checkpoint modulation that blunts genotoxic killing; and (iii) metabolic and autophagic adaptation that sustains survival under therapeutic stress (Murphy and Costa, 2020; Huang et al., 2021a; Long et al., 2026). In breast cancer, each of these layers has been causally linked to NUPR1 dependency.

Acquired resistance to endocrine agents remains a leading cause of mortality in luminal breast cancer (Chatterjee et al., 2026; Tsagkaraki et al., 2026). NUPR1 is markedly upregulated in tamoxifen-resistant ER-positive cells, it drives a clinically significant molecular subtype conversion (Bratland et al., 2000; Lee et al., 2025). Rather than maintaining a pure luminal A phenotype, these resistant cells acquire luminal B–ERBB2-positive-like characteristics, including elevated ERBB2 expression and heightened sensitivity to trastuzumab (Lee et al., 2025). Proposed mechanisms include physical interaction between NUPR1 and estrogen receptor α (ESR1) in the nucleus and co-occupancy at the promoter regions of autophagy and drug-resistance genes, which may sustain a protective autophagic shield (Elango et al., 2021; Wang et al., 2021).

Beyond autophagy maintenance, NUPR1 initiates an epigenetic bypass pathway that upregulates HER2 signaling (Lee et al., 2025). NUPR1 activates histone deacetylase 5 (HDAC5), which relieves microRNA-125a-5p-mediated repression of ERBB2, thereby elevating HER2 protein levels and promoting estrogen independence (Lee et al., 2025). This NUPR1–HDAC5–miR-125a-5p–ERBB2 axis represents a clinically actionable resistance module: targeting NUPR1 reverses the luminal-to-HER2 phenotypic switch and resensitizes cells to both endocrine and anti-HER2 interventions (Lee et al., 2025). While mechanistically compelling, alternative pathways such as the NUPR1–HDAC5–miR-125a-5p–ERBB2 epigenetic bypass pathway are supported primarily by limited cell-line studies and require broader clinical validation before their relevance can be fully established.

In TNBC, where therapeutic options are limited to cytotoxic agents, NUPR1-driven resistance is particularly pronounced (Park et al., 2021). Microfluidic selection of doxorubicin-resistant TNBC cells (L-DOXR) followed by unbiased transcriptomics identified NUPR1 as the most robustly upregulated resistance-associated gene, with expression levels strongly correlating with poor prognosis (Lim et al., 2024). This transcriptomic shift is accompanied by broad rewiring of cell-cycle, DNA-repair, and survival programs, suggesting that NUPR1 upregulation is not an isolated event but part of a system-wide adaptive response. The mechanism involves failed epigenetic control: HDAC11 is downregulated in resistant cells, leading to enrichment of H3K27 acetylation specifically at the NUPR1 promoter region 3 and relief of deacetylation-mediated transcriptional repression (Lim et al., 2024). HDAC11 inhibition reciprocally augments NUPR1 expression, confirming a direct causal relationship (Lim et al., 2024). Consequently, NUPR1 overexpression activates anti-apoptotic programs and enhances DNA-repair capacity. Beyond individual gene regulation, the HDAC11–NUPR1 epigenetic switch appears to be linked to a broader lineage plasticity program. L-DOXR cells exhibit features of the polyaneuploid cancer cell (PACC) state, suggesting that NUPR1 derepression may facilitate not merely survival but a reversible de-differentiation program that expands the pool of therapy-tolerant cells. Whether NUPR1 directly drives PACC entry through chromatin remodeling of lineage-specifying loci, or whether it primarily sustains the viability of cells already committed to this state, remains an important open question.

Under doxorubicin or paclitaxel stress, NUPR1 forms a complex with p53 and p300, binds the CDKN1A promoter, and drives nuclear accumulation of p21 together with retinoblastoma protein (RB) phosphorylation, enforcing a reversible cell-cycle arrest that allows cells to evade lethal genotoxicity (Vincent et al., 2012). This physical cooperation with the histone acetyltransferase p300 underscores that NUPR1 does not function in isolation but integrates with the epigenetic machinery to dictate cell fate decisions. By recruiting p300 to the CDKN1A locus, NUPR1 appears to favor a reversible G1/S checkpoint response over terminal apoptosis or senescence, effectively converting genotoxic stress into a transient pause that enables repair and survival. Simultaneously, NUPR1 activates PI3K/AKT signaling, inducing p21 phosphorylation and cytoplasmic translocation; this cytoplasmic p21 pool inhibits TRAIL-mediated apoptosis and reinforces chemoresistance (Vincent et al., 2012). Inhibiting NUPR1 reverses both the cell-cycle checkpoint and the anti-apoptotic blockade, resensitizing TNBC cells to anthracyclines and taxanes.

NUPR1 may further protect breast cancer cells from genotoxic agents by modulating DNA-damage repair (DDR) (Rizzuti et al., 2021). Through its chromatin-associated interactions with MSL1 and PARP1, mechanisms characterized primarily in pancreatic cancer models, NUPR1 facilitates DDR protein recruitment and restrains PARP1 enzymatic activity under homeostatic conditions (Ghandhi et al., 2014; Tang et al., 2015). In breast cancer specifically, loss of NUPR1 leads to mitochondrial dysfunction and sensitization to doxorubicin, paclitaxel, and γ-irradiation (Clark et al., 2008), although whether this sensitization is directly attributable to the NUPR1–MSL1–PARP1 axis, as opposed to other NUPR1-dependent survival pathways, remains to be fully dissected in breast cancer systems. Moreover, NUPR1 directly stimulates SUMOylation of key DDR proteins; pharmacological inhibition of NUPR1 nuclear translocation reduces SUMOylation-dependent repair functions, thereby potentiating genotoxic therapies (Lan et al., 2020). This positions NUPR1 as a synthetic-lethal node in combination with PARP inhibitors or platinum-based agents.

Moreover, ferroptosis evasion is also a resistance modality. NUPR1-mediated suppression of ferroptosis through the LCN2–iron axis underpins resistance not only to conventional cytotoxics but also to emerging ferroptosis-inducing agents (Liu and Costa, 2022; Wang et al., 2025). By maintaining iron homeostasis and redox balance, NUPR1 closes off an alternative cell-death exit that is particularly relevant in high-grade, therapy-refractory TNBC.

### Metastatic progression and microenvironmental crosstalk

Metastatic dissemination requires cancer cells to acquire both cell-intrinsic fitness for survival in circulation and the capacity to remodel distant microenvironments into permissive niches (Cetin et al., 2026; Citgez et al., 2026; Stravodimou and Voutsadakis, 2026). NUPR1 contributes to both facets through autophagy-driven bioenergetic plasticity, extracellular vesicle (EV)-mediated intercellular communication, and immunosuppressive niche engineering.

Intrinsically, the NUPR1–TFE3–autophagy axis provides the bioenergetic and biosynthetic flexibility required for migratory and invasive phenotypes (Fan et al., 2022; Wang et al., 2024). By sustaining lysosomal biogenesis and autophagic flux, NUPR1 ensures that breast cancer cells can recycle macromolecules to fuel motility and survive anoikis during transit through the vasculature (Mu et al., 2018; Wang et al., 2021). In ZR-75-30 ER-positive cells, NUPR1 knockdown inhibits not only primary tumor growth but also distant metastasis *in vivo*, phenocopying systemic autophagy inhibition (Xiao et al., 2022).

TNBC cells exploit NUPR1 packaging into tumor-derived extracellular vesicles (TEVs) to engineer a pro-metastatic niche at distant sites (Liu et al., 2024; Ortiz et al., 2024; Hong et al., 2026). Western blot and proteomic analyses demonstrate that NUPR1 is abundantly loaded into TEVs; these vesicles are transferred to recipient endothelial cells and stromal cells, where delivered NUPR1 activates pro-metastatic transcriptional programs in a type I interferon-independent manner (Ortiz et al., 2024). *In vivo*, reserpine-mediated suppression of EV release reduces NUPR1 protein levels in circulating vesicles and attenuates lung metastasis, confirming that vesicular NUPR1 is a rate-limiting factor in metastatic seeding (Ortiz et al., 2024).

NUPR1 may extend its oncogenic influence beyond tumor cells to shape the metastatic microenvironment. Pan-cancer analyses indicate that NUPR1 is highly expressed in tumor-associated macrophages (TAMs) and drives their polarization toward an M2-like, immunosuppressive phenotype in hepatocellular carcinoma, lung adenocarcinoma, and bladder cancer models (Cai et al., 2025; Ji et al., 2025; Long et al., 2026). M2-polarized TAMs secrete immunosuppressive cytokines, upregulate checkpoint molecules such as PD-L1, and induce CD8+ T-cell exhaustion, thereby dampening anti-tumor immunity and fostering a soil permissive to metastatic colonization (Zhang et al., 2025; Li and Li, 2026). We emphasize that direct evidence linking NUPR1 to M2 macrophage polarization specifically within the breast tumor microenvironment is currently lacking; the application of this mechanism to breast cancer remains hypothetical and awaits breast cancer-specific functional studies.

## Therapeutic targeting and translational opportunities

**Figure 3:**
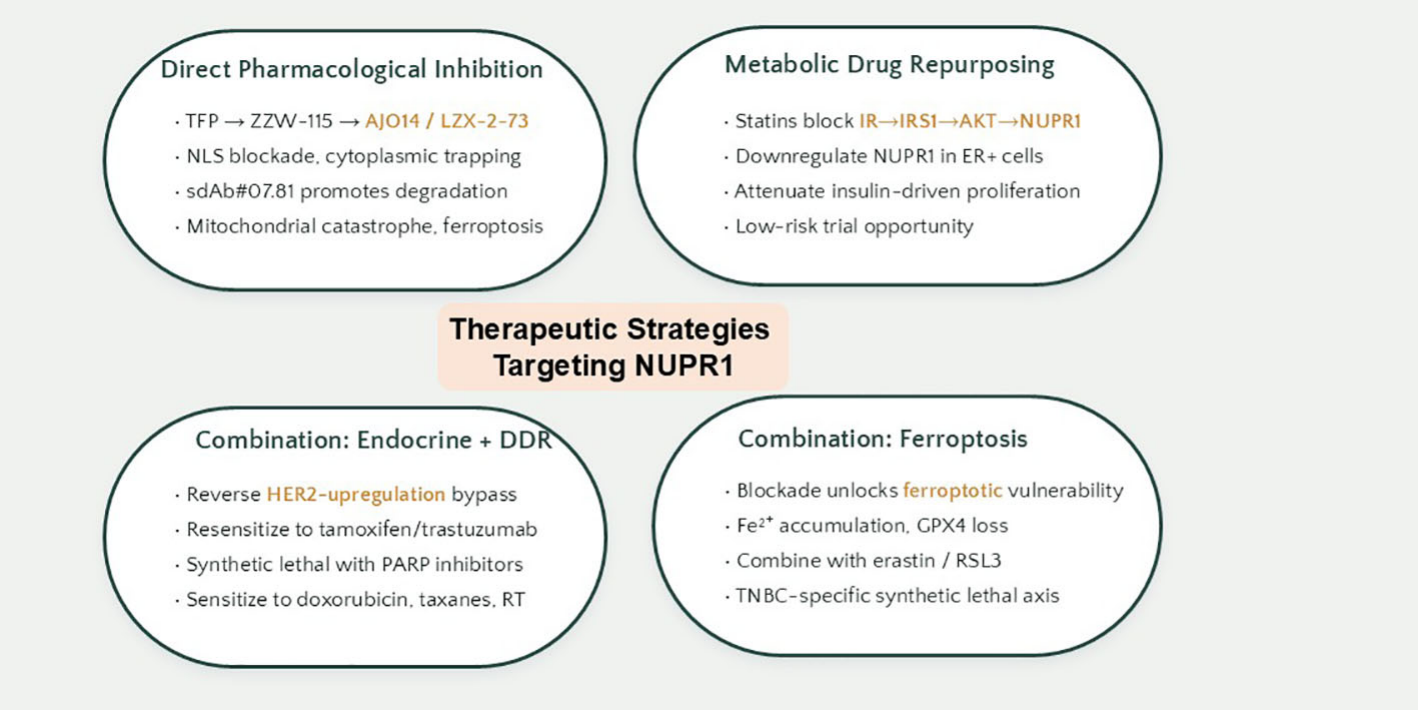
Potential therapeutic strategies targeting NUPR1.

The mechanistic architecture outlined above positions NUPR1 not merely as a biomarker of aggressive breast cancer biology but as a non-mutational, stress-adaptive dependency that can be pharmacologically dismantled to restore therapeutic vulnerability. Because NUPR1 lacks classical enzymatic activity and stable tertiary structure, its druggability historically appeared limited (Vasseur et al., 1999; Weis et al., 2015). However, recent multidisciplinary campaigns, spanning biophysics, chemical biology, and antibody engineering, have established that the conformational plasticity of this intrinsically disordered protein (IDP) can be exploited by molecules that bind to its dynamic hot spots, disrupt nuclear trafficking, and block protein–protein interactions essential for oncogenic stress responses (Deng et al., 2026; Ueno et al., 2026; Zafar et al., 2026). In this section, we synthesize the current therapeutic arsenal directed against NUPR1, examine combination strategies that align with breast cancer subtype-specific vulnerabilities, and discuss the translational trajectory required to move these agents from preclinical models to clinical practice (**Figure 3**).

### Direct pharmacological inhibition of NUPR1

The most advanced class of NUPR1 inhibitors originates from the phenothiazine scaffold of the antipsychotic trifluoperazine (TFP) (Neira et al., 2017; Santofimia-Castaño et al., 2017). Through ligand-based design and high-throughput biophysical screening, TFP was repurposed as a NUPR1 binder that interacts with the hydrophobic hot spots surrounding Ala33 and Thr68 (Santofimia-Castaño et al., 2017). Although TFP demonstrates antiproliferative activity in breast and multiple myeloma cell lines by suppressing NUPR1-driven autophagy, its clinical utility is constrained by central nervous system side effects and cardiotoxic potential via hERG channel inhibition (Murren et al., 1996; Li et al., 2017; Feng et al., 2018). To overcome these liabilities, a structure–activity optimization campaign yielded ZZW-115, a TFP-derived compound with markedly improved anticancer potency and reduced neuropsychiatric toxicity (Santofimia-Castaño et al., 2019a; Lan et al., 2020). ZZW-115 binds NUPR1 within the nuclear localization signal (NLS) region, competitively displacing importin-α family members and trapping NUPR1 in the cytoplasm (Liu et al., 2021). This blockade of nuclear translocation abrogates NUPR1-mediated transcriptional programs and simultaneously unleashes a multimodal cell death cascade (Liu et al., 2024; Santofimia-Castaño et al., 2024). Mechanistically, cytoplasmic retention of NUPR1 by ZZW-115 precipitates a mitochondrial catastrophe characterized by hyperPARylation, collapse of the mitochondrial membrane potential, depletion of ATP, and accumulation of reactive oxygen species (ROS) (Santofimia-Castaño et al., 2022; Liu et al., 2024).

Building upon the ZZW-115 scaffold, recent medicinal chemistry efforts have produced next-generation analogues. High-throughput screening of 10,000 compounds identified AJO14 as a lead that retains potent NUPR1 binding while lacking favorable affinity for the hERG potassium channel, thereby circumventing cardiotoxicity concerns (Liu et al., 2024). Further optimization generated LZX-2-73, which exhibits significantly enhanced efficacy in pancreatic and breast cancer organoids (Liu et al., 2024; Liu et al., 2025). Notably, LZX-2-73 produces strong synergistic lethality when combined with sorafenib or other kinase inhibitors, inducing massive oxidative stress, lipid peroxidation, and caspase activation (Liu et al., 2025). These advances provide a preclinical rationale for NUPR1 small-molecule inhibitors, suggesting that such compounds could potentially be refined to improve pharmacodynamic potency while eliminating off-target liabilities, and rationally paired with existing targeted agents to explore synthetic lethal interactions.

Beyond conventional small molecules, the structural openness of NUPR1 has recently enabled a biologic targeting approach. Using an integrated high-throughput platform combining *in situ* proximity ligation assay followed by DNA sequencing (isPLA-seq), NanoBiT proximity assays, and C-degron degradation validation, investigators have screened large single-domain antibody (sdAb) libraries and identified sdAb#07.81 as a lead candidate (Wang et al., 2025). This sdAb exhibits strong binding affinity for NUPR1 and, upon intracellular delivery, promotes the degradation of endogenous NUPR1 in triple-negative breast cancer (TNBC) cells (Wang et al., 2025). Functional characterization demonstrates that sdAb#07.81 suppresses malignant proliferation, induces premature senescence, and derepresses ferroptosis (Wang et al., 2025). In fully immunocompetent murine 4T1-derived models, systemic delivery of sdAb#07.81 significantly reduces tumor burden, lowers NUPR1 protein levels, and diminishes proliferative indices (Wang et al., 2025). This proof-of-concept establishes that intracellular biologics can effectively neutralize an IDP oncogene, opening a new modality for patients whose tumors exhibit high NUPR1 expression and limited druggable kinase targets.

Despite these promising preclinical findings, substantial limitations remain. NUPR1 is an intrinsically disordered protein that lacks deep hydrophobic pockets, making structure-based optimization difficult and likely limiting the maximal binding affinity of current small molecules to the micromolar range. Furthermore, phenothiazine-derived scaffolds retain a well-documented risk of hERG channel inhibition and central nervous system penetration, while normal tissues with high basal NUPR1 expression may be susceptible to on-target toxicity. Finally, no NUPR1-targeted small molecule or biologic has yet entered phase I clinical trials for breast cancer, underscoring the early translational stage of this strategy.

### Metabolic drug repurposing

An alternative, immediately translatable strategy involves repositioning widely prescribed metabolic agents to indirectly impinge upon the NUPR1 axis. In obesity-associated, hyperinsulinemic breast cancer, the insulin receptor (IR)–IRS1–AKT signaling cascade robustly induces NUPR1 expression and stabilizes the protein via PEST-domain phosphorylation, creating a feed-forward loop that fuels oncogenic metabolism (Scordamaglia et al., 2026). Statins, lipid-lowering drugs that inhibit HMG-CoA reductase and the mevalonate pathway, have been shown to impair IR-mediated signaling and downregulate NUPR1 expression in ER-positive breast cancer cells (Alim and Akalanka, 2026; Scordamaglia et al., 2026). Mechanistically, simvastatin, atorvastatin, and rosuvastatin attenuate insulin-stimulated proliferation and clonogenic capacity in MCF7 and high-IR-expressing BCAHC-1 cells, and this cytostatic effect is rescued by ectopic NUPR1 overexpression (Alim and Akalanka, 2026). Because hyperinsulinemia and dyslipidemia are prevalent comorbidities in breast cancer patients, and because statins are already approved with established safety profiles, clinical trials evaluating statin co-administration in NUPR1-high, metabolically dysregulated luminal disease represent a low-risk, high-yield translational opportunity (Mauriello et al., 2025; El Sayed, 2026). This approach exemplifies how understanding the metabolic–stress interface of NUPR1 can convert epidemiological observations into mechanism-based therapeutic regimens. Nevertheless, this repurposing strategy faces notable constraints. The magnitude of NUPR1 suppression achieved by statins in preclinical models is modest, and whether the reduction in NUPR1 expression is sufficient to blunt insulin-driven oncogenesis in patients remains uncertain. Additionally, the pleiotropic effects of statins, including anti-inflammatory and anti-angiogenic activities, confound the attribution of clinical benefit specifically to NUPR1 inhibition. Retrospective epidemiological studies of statins in breast cancer have yielded inconsistent results, and no prospective trial has stratified patients by tumor NUPR1 status, leaving the biomarker-driven hypothesis formally untested.

### Combination strategies targeting resistance mechanisms

The multifaceted resistance architecture governed by NUPR1, spanning endocrine persistence, DNA-damage repair, ferroptosis evasion, and immune suppression, provides a rational framework for combination therapies. In luminal A breast cancers that acquire tamoxifen resistance, NUPR1 drives a deleterious phenotypic switch toward luminal B–ERBB2-positive-like characteristics by activating HDAC5 and derepressing ERBB2 via the miR-125a-5p axis (Lee et al., 2025). Pharmacological inhibition of NUPR1 by ZZW-115 or genetic depletion disrupts the NUPR1–ESR1 transcriptional complex, may collapse the protective autophagic program, and reverse the HER2-upregulation bypass. Consequently, NUPR1 blockade may resensitize tamoxifen-resistant cells to both endocrine deprivation and trastuzumab (Lee et al., 2025).

NUPR1 directly participates in DNA-damage repair through its chromatin-associated interactions with MSL1 and PARP1 (Gironella et al., 2009). Under homeostatic conditions, NUPR1 restrains PARP1 enzymatic activity; upon genotoxic insult, NUPR1 facilitates the recruitment of repair proteins and stimulates SUMOylation of DNA-damage response (DDR) factors. Inhibiting NUPR1 nuclear translocation with ZZW-115 down-regulates the SUMOylation of core DDR factors, effectively lowering the genotoxic threshold of triple-negative breast cancer cells (Lan et al., 2020).This chemosensitization strategy provides a promising therapeutic window for pairing with advanced drug delivery systems, such as precisely tailored doxorubicin prodrug nanoassemblies, which can exploit these newly exposed vulnerabilities to maximize intratumoral activation while limiting systemic toxicity (Feng et al., 2024). Moreover, the hyperPARylation and mitochondrial catastrophe induced by NUPR1 inactivation create a synthetic lethal interaction with PARP inhibitors and platinum-based agents, particularly in high-grade TNBC and BRCA-deficient contexts (Santofimia-Castaño et al., 2022; Iovanna et al., 2025).

NUPR1 is a canonical repressor of ferroptosis through its transcriptional upregulation of LCN2 and FTH1, which limit labile iron availability and lipid peroxidation (Liu et al., 2021; Tan et al., 2024). In TNBC, where apoptotic plasticity is limited and iron dependence is high, NUPR1 blockade unlocks ferroptotic vulnerability. Pharmacological inhibition of NUPR1 with ZZW-115, or genetic silencing, precipitates intracellular Fe^2+^ accumulation, glutathione depletion, GPX4 downregulation, and ACSL4-mediated lipid peroxide generation (Liu and Costa, 2022; Tan et al., 2024; Wang et al., 2025). These biochemical changes are fully rescued by ferrostatin-1 or iron chelators, confirming pathway specificity. Combining NUPR1 antagonists with ferroptosis inducers such as erastin or RSL3 may provide a potent therapeutic axis for TNBC and other NUPR1-high, iron-dependent breast cancer subtypes (Deng and Li, 2026; Iftikhar et al., 2026; Lin et al., 2026).

Practical execution of these combinations is fraught with unresolved challenges. Overlapping toxicities may preclude full-dose concurrent administration, yet optimal sequencing and dosing schedules have not been explored even in preclinical models. Moreover, NUPR1 inhibition may trigger compensatory resistance via NRF2-driven antioxidant programs or alternative iron-export pathways, and the safety of pairing ferroptosis inducers with NUPR1 antagonists has not been assessed in normal tissue models. Regulatory and co-development complexities for such novel combination regimens will require careful navigation before clinical testing can be contemplated.

## Conclusions and perspectives

NUPR1 has emerged as a central, non-mutational factor implicated in stress adaptation in breast cancer, with evidence linking it to metabolic insults, therapeutic pressure, and malignant survival. As an intrinsically disordered protein, it exploits extreme conformational plasticity to integrate diverse oncogenic signals, including insulin-driven mitogenic stimuli, DNA damage, and nutrient deprivation, into coherent transcriptional and metabolic outputs. Its capacity to function as a chromatin-associated co-regulator, a cytoplasmic scaffold for stress granules, and a gatekeeper of autophagic and ferroptotic circuits positions NUPR1 not merely as a biomarker of aggressive disease, but as a functional dependency co-opted systematically across molecular subtypes.

The molecular architecture of NUPR1 underpins this versatility. The interplay between its N-terminal PEST domain, C-terminal NLS/HLH module, and dynamic post-translational modification landscape creates a regulatory rheostat that couples growth-factor signaling to subcellular localization and interactome selection. This plasticity enables context-dependent engagement with NF-κB, SMAD, PI3K/AKT, and ESR1 axes, allowing NUPR1 to tailor its outputs to luminal, HER2-enriched, or triple-negative contexts.

Pathobiologically, NUPR1 sits at the nexus of metabolic reprogramming, proteostatic control, and cell-fate determination. By transducing insulin–PI3K/AKT signaling into glycolytic programs, sustaining TFE3-driven autophagic flux, and suppressing ferroptosis through LCN2-mediated iron export and ferritin sequestration, NUPR1 constructs multi-layered defenses against microenvironmental stress and cytotoxic therapy. Its roles in endocrine resistance via ESR1 cooperation, chemoresistance through p21-mediated checkpoint control and DDR SUMOylation, and metastatic dissemination through extracellular vesicle-mediated niche engineering underscore its multidimensional threat to therapeutic efficacy. These mechanisms collectively establish NUPR1 as a synthetic-lethal node whose pharmacological dismantling could collapse compensatory survival networks across treatment modalities.

Therapeutically, targeting NUPR1 has transitioned from theoretical challenge to tangible clinical opportunity. Small-molecule inhibitors including ZZW-115, AJO14, and LZX-2-73—optimized to disrupt NUPR1–importin-α interactions—demonstrate that the dynamic hot spots of intrinsically disordered proteins are pharmacologically tractable. These agents unleash multimodal cell death while sensitizing tumors to genotoxic agents, PARP inhibitors, and anti-HER2 therapies. The single-domain antibody sdAb#07.81 provides proof-of-concept that intracellular biologics can promote endogenous NUPR1 degradation and alleviate TNBC progression, opening a new modality for patients with limited druggable targets. Concurrently, statin-mediated suppression of the insulin–NUPR1 axis offers an immediately translatable strategy to intercept obesity-associated, hyperinsulinemic breast cancer using agents with established safety profiles.

Several challenges remain. First, robust, subtype-specific biomarkers are needed to stratify patients most likely to benefit from NUPR1 inhibition. Second, the optimal integration of NUPR1 antagonists into existing paradigms requires rigorous clinical validation. Third, pharmacokinetic and intracellular delivery barriers facing biologic modalities necessitate innovative formulation strategies. Fourth, deeper structural understanding of NUPR1 phase separation dynamics and isoform-specific conformational ensembles will be essential for rational drug design.

Looking forward, we envision a precision-oncology framework in which NUPR1 expression, modification status, and subcellular localization guide therapeutic decision-making. Future research should leverage single-cell and spatial multi-omics to dissect NUPR1 heterogeneity within tumor ecosystems, particularly its crosstalk with tumor-associated macrophages and endothelial cells. Translating NUPR1 antagonists into clinical trial design requires moving past traditional genomic selection models. Because NUPR1 operates as a non-mutational dependency, routine next-generation sequencing panel assays will fail to identify eligible patients. Instead, patient stratification strategies must utilize validated quantitative immunohistochemistry (IHC) platforms designed to evaluate shifting nucleocytoplasmic localization patterns, or rely on machine-learning-derived multi-gene transcriptomic signatures that capture downstream pathway activation. Exploring NUPR1’s role in immunomodulation beyond macrophage polarization may reveal opportunities for combination with immunotherapy. Conceptually, dismantling the stress-adaptive network associated with NUPR1 could represent a paradigm shift from targeting static, mutation-driven oncogenes to exploiting dynamic, non-mutational dependencies. Sustained interdisciplinary collaboration will be required, but the reward—converting a universal stress-adaptive node into a clinically actionable, precision vulnerability—promises to be substantial.
